# Feasibility and Safety of Fiber Optic Micro-Imaging in Canine Peripheral Airways

**DOI:** 10.1371/journal.pone.0084829

**Published:** 2014-01-09

**Authors:** Yijun Liu, Bingbing Yan, Ziyang Huang, Rui Guo, Jingxing Wu, Xun Liu, Kaiqing Yao, Fajin Lv, Huisheng Deng

**Affiliations:** 1 Department of Geriatrics, The First Affiliated Hospital of Chongqing Medical University, Chongqing, China; 2 Department of Critical Care Medicine, The First Affiliated Hospital of Chongqing Medical University, Chongqing, China; 3 Department of Radiology, The First Affiliated Hospital of Chongqing Medical University, Chongqing, China; 4 Department of Respiratory, The First Affiliated Hospital of Chongqing Medical University, Chongqing, China; University of California, Merced, United States of America

## Abstract

**Purpose:**

To assess the feasibility and safety of imaging canine peripheral airways (<1 mm) with an experimental micro-imaging fiber optic bronchoscope.

**Methods:**

Twenty healthy dogs were scoped with a micro-imaging fiber optic bronchoscope (0.8 mm outer diameter). Images at various levels of the bronchioles, mucosal color, and tracheal secretions were recorded. The apparatus was stopped once it was difficult to insert. CT imaging was performed simultaneously to monitor progression. The safety of the device was evaluated by monitoring heart rate (HR), respiratory rate (RR), mean artery pressure (MAP), peripheral oxygen saturation (SpO_2_) and arterial blood gases (partial pressure of arterial carbon-dioxide, PaCO_2_, partial pressure of arterial oxygen, PaO_2_, and blood pH).

**Results:**

(1) According to the CT scan, the micro-imaging fiber was able to access the peripheral airways (<1 mm) in canines. (2) There was no significant change in the values of HR, MAP, pH and PaCO_2_ during the procedure (P>0.05). Comparing pre-manipulation and post-manipulation values, SpO_2_ (F = 13.06, P<0.05) and PaO_2_ (F = 3.01, P = 0.01) were decreased, whereas RR (F = 3.85, P<0.05) was elevated during the manipulation. (3) Self-limited bleeding was observed in one dog; severe bleeding or other complications did not occur.

**Conclusion:**

Although the new apparatus had little effect on SpO_2_, PaO_2_ and RR, it can probe into small peripheral airways (<1 mm), which may provide a new platform for the early diagnosis of bronchiolar diseases.

## Introduction

Although bronchiolar diseases, including acute bronchiolitis and diffuse panbronchiolitis, are not closely tracked in China, they are common and difficult to diagnose. This limits the progress of research and threatens human life and health. Hence, there is an increasing need to evaluate and manage bronchiolar diseases.

With the development of imaging modalities, both computed tomography (CT) and positron emission tomography (PET) are playing a greater role in the evaluation of peripheral airways. CT scans are very useful but lack the sensitivity and specificity necessary to definitively establish a diagnosis on its own [1], [2]. Similarly, although PET is more sensitive than CT in identifying lesions in the peripheral segments of the lung, there are too many false positives to allow treatment decisions to be consistently made based on PET imaging alone [1], [2]. It is also too expensive to widely apply in clinic, especially in developing countries.

The diagnosis of a central pulmonary lesion still relies on pathology results. This allows for a clear diagnosis and pathological classification, which can guide treatment. Bronchofiberscopes of different diameters have been used to evaluate solitary pulmonary nodules and masses for more than 30 years. Patients with such nodules frequently undergo transbronchial biopsy under fluoroscopic guidance. However, endoscopic information often fails to successfully detect peripheral airways smaller than 2 mm in diameter [3]. In addition, due to challenging technology and the potential for severe complications, thoracoscopy is not commonly used as a diagnostic tool alone [4], [5].

These diagnostics play a key role in treatment decisions, but they are poor at inspecting small lesions (<1 mm). Direct viewing of the peripheral airways would provide higher-level endoluminal information for the diagnosis of bronchiolar diseases, such as acute bronchiolitis, bronchioloalveolar carcinoma, occlusive bronchiolitis, follicular bronchiolitis, diffuse panbronchiolitis, childhood asthma, and mineral dust airways diseases [6]. Therefore, a new instrument was urgently needed to address these issues. The current study was designed to assess the feasibility and safety of viewing the peripheral airways (<1 mm) in canines with a micro-imaging fiber optic bronchoscope (0.8 mm).

## Materials and Methods

### Experimental Apparatus

**Figure 1.View:**
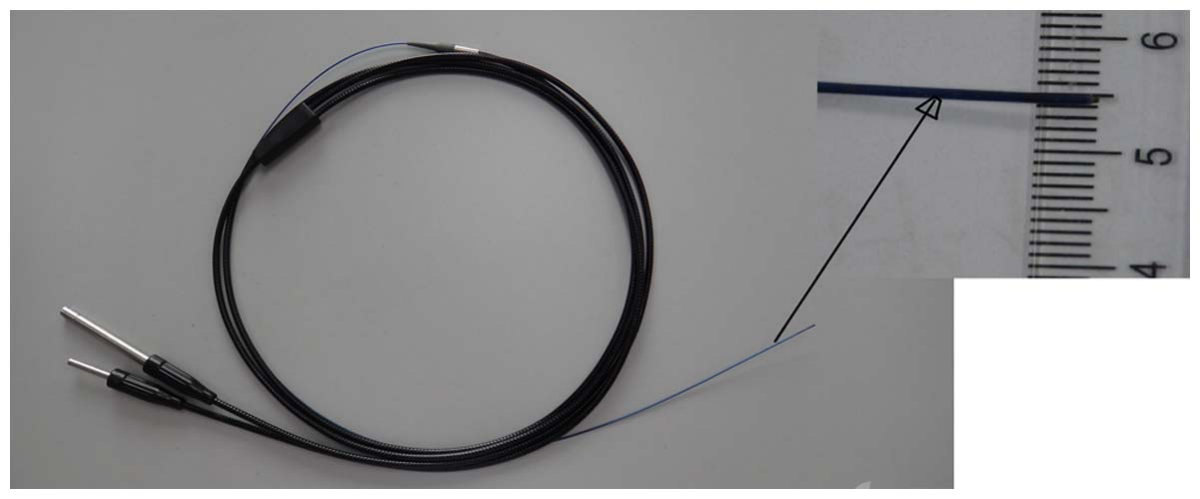
of the entire micro-imaging fiber, showing the body of the fiberscope, objective lens, and connection to camera.

The device included a flexible micro-imaging fiber optic bronchoscope (FVS-001MI, Blade Co., Beijing, China) and a computer processor and monitor. The OD of the bronchoscope was 0.8 mm (Fig. 1), and the distal end could bend between 0° and 60°. The total length of the scope was 2440 mm; the length of the working portion was designed to be 560 mm for accessing the complicated tracheobronchial tree. It had a visual angle of 75 degrees, and a range of observation of 3 mm to 30 mm. The other specifications are as previously reported [7]. With the light source attached, the scope recorded real-time images in the airways, and the signals were processed and displayed on a screen.

### Ethics Statement

This study was carried out in strict accordance with the recommendations in the Guide for the Care and Use of Laboratory Animals of the National Institutes of Health. The protocol was approved by by the Medical Animal Care & Welfare Committee of the First Affiliated Hospital of Chongqing Medical University, Chongqing, China (Permit Number: 2013–004). All procedures were performed under sodium pentobarbital anesthesia, and all efforts were made to minimize suffering.

### Animals and surgical procedures

Twenty healthy dogs of either gender (15–20 kg) were used in this study. After 12 hours of fasting, all the canines were placed in the supine position. The animals were anesthetized by intraperitoneal injection of pentobarbital using a dosage of 30 mg/kg body weight. During the experiment, small doses of pentobarbital (2–3 mg/kg body weight) were given each hour in order to provide an adequate depth of anesthesia.

Immediately after induction of anesthesia, an endotracheal tube (Mallinckrodt Medical Co. Ltd., Co. Westmeath, Ireland, ID = 7.5 mm) was inserted to permit mechanical ventilation and to facilitate bronchoscopy. Respiratory rate (RR, breaths per minute), heart rate (HR, beats per minute), mean arterial pressure (MAP, mmHg), SpO_2_(%), and arterial blood gases (ABG) were recorded 5 minutes (min) before each operation, at 5 min intervals during the manipulation and at 5 min and 10 min after the manipulation. All arterial blood samples taken from the right femoral artery were immersed in ice and measured within 30 minutes on a blood gas auto-analyzer at 37°C.

### Micro-imaging fiber manipulation

All canines were imaged and photographed by a trained bronchoscopist from oral cavity to small peripheral airways. If the experimenter observed a large amount of secretions in the central airway, which heavily affected visualization, then the visual sputum suctioning system [7] was used to clean the visual field. The signal was recorded on the computer with an acquisition system. During manipulation, if the blood pressure decreased by more than 20% of the initial blood pressure, ephedrine (5 mg) was administered. If bleeding was observed, the operator stopped the procedure, avoiding further damage to the bronchioles. Since all dogs were healthy and should have normal coagulation, bleeding may be due to damage of the capillary membranes generated by the instrument or operator error. Therefore, bleeding should stop immediately upon aborting the procedure. At the end of each experiment, the airway device was removed, and the dogs were euthanized with an overdose of sodium pentobarbital.

### Statistics

SPSS software (Version 21.0, SPSS Ltd., Chicago, Illinois, US) was used for data analysis, and results were expressed as mean ± standard deviation (SD). The safety was performed as an analysis of variance (ANOVA). A p value<0.05 was considered to be statistically significant.

## Results

The experiments were well tolerated, and vital signs were stable throughout the procedures. Every experiment required 30–40 minutes starting at the time of intubation. We encountered no severe problems associated with using the device; no canine suffered any major complication from the procedure. Self-limited bleeding was observed in one dog; severe bleeding did not occur. There were no other complications. The scope was as easy to maneuver as a standard size adult bronchoscope.

### CT and Micro-imaging fiber findings

**Figure 2:**
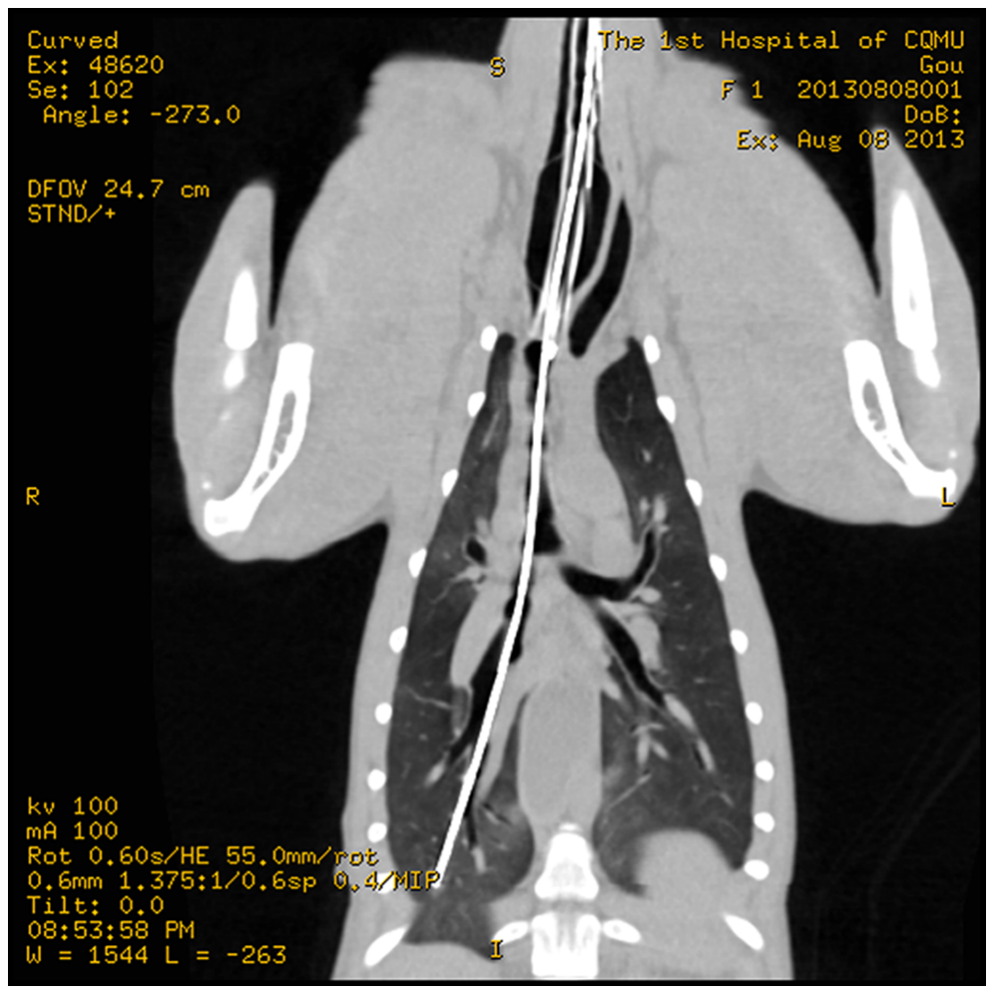
The tip of the micro-imaging fiber extending to the peripheral bronchioles in a canine.

**Figure 3:**
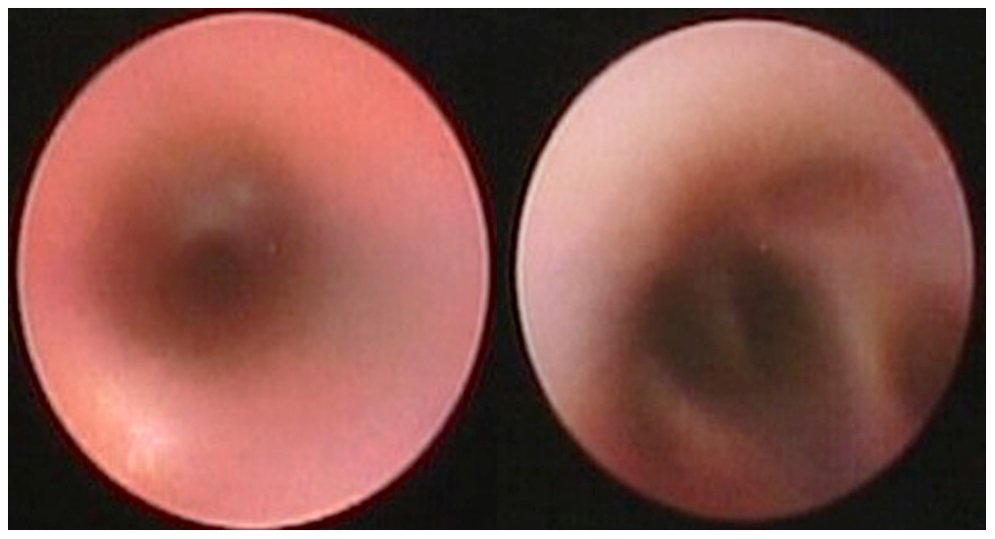
Real-time imaging provides peripheral bronchial intraluminal information and peripheral bronchial branches in a healthy canine.

Under real-time imaging guidance, the micro-imaging fiber optic bronchoscope was able to enter peripheral airways less than 1 mm in diameter (video S1) (Fig. 2). In all the dogs, the central airways contained a large amount of secretions. Similarly, the peripheral airways also contained a mild amount of white foamy secretions. The mucosa of the peripheral airways had a smooth and shiny surface, was pale reddish, and part of the color was not uniform (Fig. 3).

### Safety study

Comprehensive changes in basic physiologic parameters were recorded in all 20 animals. Table S1 shows the effects on HR, RR, SpO_2_, MAP and arterial blood gases during the experimental period. There was no significant change in HR, MAP, pH or PaCO_2_ during the procedure (P>0.05). Comparing pre-manipulation and post-manipulation, SpO_2_ (F = 13.06, P<0.05) and PaO_2_ (F = 3.01, P = 0.01) were decreased, but RR (F = 3.85, P<0.05) was elevated during the manipulation.

## Discussion

### Summary of the main findings

The main results of the study were as follows: (1) According to the CT scan, the micro-imaging fiber optic bronchoscope was able to image the peripheral airways (<1 mm) of dogs. (2) There was no significant change in HR, MAP, pH and PaCO_2_ during the procedure (P>0.05). Comparing pre-manipulation and post-manipulation, SpO_2_ (F = 13.06, P<0.05) and PaO_2_ (F = 3.01, P = 0.01) were decreased, however, RR (F = 3.85, P<0.05) was elevated during the manipulation. (3) Self-limited bleeding was observed in one dog; severe bleeding did not occur.

### Significance of diagnosis for peripheral airways

Anatomically, bronchioles are small airways (internal diameter of 2 mm or less) that do not contain cartilage in their walls; they are accompanied by branches of the pulmonary artery and include membranous, terminal and respiratory bronchioles [8], [9]. Because bronchioles are between bronchial and lung parenchyma, lesions in these areas are often left untreated until they become severe, and large airway and parenchymal lung lesions are affected. Bronchioles are anatomically different from central airways in many ways [10]: 1) there is no cartilage and hardly any bronchial glands; 2) the muscular tunic is well developed as compared with lumen; 3) there are few ciliated and goblet cells. In addition, in the normal lung bronchioles contribute little to airway resistance, but when diseased can have a major impact on lung function. In fact, patients with severe clinical symptoms may exhibit relatively minor pathological changes. Because of these differences, there should be distinctions between endoscopic findings in peripheral airways and those in central airways. Additionally, some diseases, such as occlusive bronchiolitis, follicular bronchiolitis, and diffuse panbronchiolitis, commonly occur in the bronchioles less than 2 mm [11] and may affect bronchioles less than 1 mm in diameter. These diseases are common and hard to diagnose early, which is a threat to human life and health.

### A comparison of micro-imaging fiber optic bronchoscopes and current endoscopes

Due to the special anatomy, the diagnosis of the peripheral pulmonary small lesions via the common bronchoscope is challenging. Although thoracoscopy is able to determine a clear diagnosis and make pathological classifications of these lesions, it requires specialized technology and has high risk, even mortality [4]. This is less likely to be used as the sole diagnostic instrument. Studies have shown that ultrathin bronchoscopes can detect small airways as far as the 12th or 13th generation (about 2 mm) [12], [13], but it is hard to provide higher-level information for diagnosis of bronchiolar diseases, such as acute bronchiolitis, bronchioloalveolar carcinoma, occlusive bronchiolitis, follicular bronchiolitis, diffuse panbronchiolitis, childhood asthma, and mineral dust airways diseases. Therefore, it is important to produce a new device, which can be used to directly examine the so-called “silent” or “quiet” zone [14], and could potentially provide new information for the diagnosis of bronchiolar diseases.

Rapid progress in optical fiber technology has reduced the diameter of the micro-imaging fiber to 0.8 mm, creating this promising new apparatus. As the OD of the new device used in this study is 0.8 mm, it can now obtain previously unobtainable information by direct imaging of the peripheral airways.

To the best of our knowledge, this is the first report to demonstrate the application of imaging guidance for detecting bronchioles using an optical fiber of such diameter. However, fiber optic ductoscopy has previously been described as a diagnostic tool for nipple discharge [15], [16]. One limitation of this instrument is the image resolution of the micro-imaging fiber is 6000 pixels, which is about one fifth less than that of other endoscopes [17], which should be improved in future. However, this scope was as easy to maneuver as a standard size adult bronchoscope.

### Safety evaluation

In this study, the manipulation caused a sustained decrease in PaO_2_, SpO_2_ and an elevation in RR. Several reasons are suspected for this phenomenon. First, when the instrument (OD = 0.8 mm) was placed, the cross section of the airway decreased. Second, a reflex induced by mechanical stimulations of the airway could have lead to bronchoconstriction [18]. Therefore, the increasing RR could have compensated for hypoxia.

During the procedure, although there was no significant change in HR, MAP and PaCO_2_, and no PaO_2_ values less than 60 mmHg, the safety should be further assessed. In addition, future research should use clinical trials to determine whether this system has additional safety benefits.

### Clinical perspective

In addition to the benefits of diagnosing primary and secondary peripheral airway disease, this technology could be further integrated into other medical technologies. A micro-imaging fiber has been developed in our previous research, which incorporated the micro-imaging fiber into a conventional catheter and developed a visual sputum suction system. In addition, the new scope was incorporated into an existing puncture device and established a Visual Puncture System, which provides a new instrument platform for the puncture and drainage of thoracic, abdominal, and pericardial fluid.

## Limitations

The apparatus, however, has several functional drawbacks including difficulty observing the field due to flexibility and difficulty identifying the generation of bronchial branches in the upper lungs. In most cases, the apparatus approached the peripheral airways in the lower lobe lungs. Furthermore, biopsies of the abnormalities observed could not be performed at this time, so the morphological changes of the peripheral airways could not be pathologically assessed. However, this could be improved by adding a biopsy channel. In addition, the instrument can cause self-limited bleeding due to damaging the capillary membranes, but this can be limited by user training and modifying the design.

## Conclusion

In conclusion, the new apparatus has little effect on PaO_2_, SpO_2_ and RR, but this drawback should be compensated. The new device, however, can probe into small peripheral airways. Direct observations can reach levels less than 1 mm in diameter. Bronchiolar images were comprehensive, and color photographs can be taken. For these reasons, this micro-imaging fiber optic bronchoscope shows considerable promise for wide application obtaining endoluminal information and early diagnosis in the peripheral segments of the lung, especially as a complement to conventional methods.

## Supporting Information

Table S1 **Changes in physiological parameters at various times (pre-op, intra-op and post-op) in canines (mean ± SD).** pre-op  =  pre-operation, intra-op  =  intra-operation, post-op  =  post-operation. _*_ p values were^ significantly different from that of^ pre-operation and _post-_operation.
(DOC)

Video S1 **Real-time imaging guidance using the micro-imaging fiber optic bronchoscope in a dog.** The video shows the micro-imaging fiber entering the peripheral bronchioles less than 1 mm in diameter.
(AVI)
