# Supplementary material for: Feasibility and Safety of Fiber Optic Micro-Imaging in Canine Peripheral Airways
Source: PLoS One. 2014 Jan 9;9(1):e84829. doi: 10.1371/journal.pone.0084829 (PMC3886988; doi:10.1371/journal.pone.0084829)
Supplement: Table S1 — Changes in physiological parameters at various times (pre-op, intra-op and post-op) in canines (mean ± SD). pre-op = pre-operation, intra-op = intra-operation, post-op = post-operation. * p values were significantly different from that of pre-operation and post-operation. (DOC) [file pone.0084829.s001.doc]

|  | HR  （beats per minute) | SpO_2_  （%） | RR  （breaths per minute） | MAP  (mmHg) | PaCO_2_  （mmHg） | PaO_2_  （mmHg） | pH |
| --- | --- | --- | --- | --- | --- | --- | --- |
| pre-op | 114.35±14.04 | 96.25±3.18 | 22.40±4.39 | 86.45±6.57 | 40.85±4.66 | 89.45±5.17 | 7.38±0.06 |
| intra-op | | | | | | | |
| 5 min | 114.20±17.26 | 89.90±4.24* | 25.65±4.11* | 88.10±8.94 | 42.35±5.58 | 84.95±6.54* | 7.36±0.05 |
| 10 min | 115.10±18.60 | 90.15±4.45* | 26.15±4.40* | 87.95±7.85 | 40.95±4.38 | 84.55±7.12* | 7.35±0.07 |
| 15 min | 118.05±16.59 | 89.35±5.16* | 25.40±4.50* | 87.63±9.64 | 42.05±4.84 | 85.20±7.22* | 7.36±0.07 |
| post-op | | | | | | | |
| 5 min | 118.05±15.41 | 95.25±2.92 | 22.25±4.30 | 88.20±7.57 | 41.05±3.44 | 89.50±6.76 | 7.36±0.05 |
| 10 min | 115.45±22.00 | 94.95±2.63 | 22.10±4.60 | 88.15±7.92 | 40.40±2.85 | 89.40±5.65 | 7.37±0.05 |
